# Analysis of the dislocation activity of Mg–Zn–Y alloy using synchrotron radiation under tensile loading

**DOI:** 10.1107/S1600577523003491

**Published:** 2023-05-11

**Authors:** Changwan Ha, Young Min Kim, Sang Kyu Woo, Emad Maawad, Dietmar Letzig, Sangbong Yi

**Affiliations:** a Institute of Material and Process Design, Helmholtz-Zentrum Hereon, Geesthacht, Germany; b Department of Magnesium, Korea Institute of Materials Science, Changwon, Republic of Korea; c Institute of Material Physics, Helmholtz-Zentrum Hereon, Geesthacht, Germany; University of Malaga, Spain

**Keywords:** dislocation, slip activity, synchrotron radiation, tensile test, magnesium alloy

## Abstract

An understanding of deformation behavior and texture development is crucial for the formability improvement of Mg alloys. X-ray line profile analysis using the convolutional multiple whole profile (CMWP) fitting method allows the experimental determination of dislocation densities separately for different Burgers vectors up to a high deformation degree. A wider use of this technique still requires exploration and testing of various materials. In this regard, the reliability of the CMWP fitting method for Mg–Zn–Y alloys, in terms of the dislocation activity during tensile deformation, was verified in the present study by the combined analysis of electron backscatter diffraction (EBSD) investigation and visco-plastic self-consistent (VPSC) simulation. The predominant activity of non-basal 〈*a*〉 dislocation slip was revealed by CMWP analysis, and Schmid factor analysis from the EBSD results supported the higher potential of non-basal dislocation slip in comparison with basal 〈*a*〉 dislocation slip. Moreover, the relative slip activities obtained by the VPSC simulation also show a similar trend to those obtained from the CMWP evaluation.

## Abstract 2

The convolutional multiple whole profile fitting method of X-ray line profile analysis using synchrotron radiation allows the experimental determination of dislocation densities separately for different Burgers vectors up to a high degree of deformation. Its reliability for Mg–Zn–Y alloys, in terms of the dislocation activity during tensile deformation, has been successfully verified by combined analysis with electron backscatter diffraction investigation and visco-plastic self-consistent simulation.

## 1. Introduction

Wrought Mg alloy sheets have an issue of low formability, which is due to the limited number of active deformation modes and the formation of a strong crystallographic texture during thermomechanical treatments. The alloying addition of rare-earth (RE) elements or Ca affects the microstructure and texture development in Mg alloy, and has been acknowledged as an appropriate method to overcome the low formability at room temperature (Bohlen *et al.*, 2007 ▸, 2015 ▸; Chino *et al.*, 2011 ▸). It has been verified that analysis of the deformation behavior and texture development plays an important role in the improvement of formability. For that reason, research on Mg has led to a comprehensive understanding of its deformation behavior, *e.g.* activities of dislocations slip and formability at ambient temperature in relation to the initial textures of various alloy systems.

Regarding the deformation mechanisms of Mg sheets, the activities of non-basal 〈*a*〉 and pyramidal 〈*c*+*a*〉 dislocation slip systems have been reported using electron microscopy observations, *i.e.* SEM or TEM (scanning- or transmission electron microscope) and/or computer simulations based on dislocation and elastoplastic polycrystal models of deformation and texture (Sandlöbes *et al.*, 2012 ▸; Agnew *et al.*, 2003 ▸, 2006 ▸). TEM observation directly provides the active deformation modes with visual images. It is, however, limited to a small volume and strain, and an enormous exertion is required, especially to observe *in situ* dislocation activities during deformation owing to a large strain. The results from numerical approaches, *e.g.* the VPSC (visco-plastic self-consistent) model, are largely dependent on the correct approximation of the initial parameters, and the precise data of stress–strain curves and the evolution of deformation texture are needed to provide qualified information for determination of the initial parameters. On the other hand, X-ray line profile analysis using *in situ* hard X-ray diffraction provides information on the deformation in a relatively large sample volume due to its high penetration depth. Furthermore, it provides remarkable resolution for the analysis of high dislocation density, even at relatively high strain levels. Simultaneously, the sample preparation for such *in situ* X-ray line profile analysis is relatively simple, due to non-destructive testing, and there is no sample damage caused by the irradiation. The CMWP (convolutional multiple whole profile) fitting method from X-ray line profile analysis has been applied to analyze specific dislocation slip systems based on well established physical principles in crystalline materials (Dutta *et al.*, 2021 ▸; Drozdenko *et al.*, 2021 ▸; Ha *et al.*, 2021 ▸). However, to be widely used for the CMWP fitting method, the efforts of many researchers and the application of various materials is still required. In addition, the reliability of the X-ray line profile analysis using the CMWP fitting method needs to be verified by different methods, *e.g.* comparative studies with electron microscopy and crystal plasticity simulation, while it has great potential for the quantitative characterization of lattice defects (Dragomir & Ungár, 2002 ▸; Máthis *et al.*, 2004 ▸, 2015 ▸; Ribárik *et al.*, 2020 ▸).

The aim of the present study is to evaluate the dislocation activity using the CMWP fitting method during tensile deformation beyond the early stage of plastic deformation of Mg–Zn–Y alloy sheets. From *in situ* diffraction experiments using synchrotron radiation, the development of deformation texture and tracking of individual dislocation slip systems with different Burgers vectors could be obtained. The obtained results were assessed comparatively with the data achieved by SEM observation and VPSC simulation and the reliability of the CMWP fitting method was verified.

## 2. Experimental

ZW10 (Mg-0.96 wt% Zn-0.38 wt% Y) alloy was gravity cast at 715°C into a rectangular steel mold and was machined as a slab with a thickness of 10 mm. Homogenization annealing was carried out at 420°C for 16 h prior to the rolling process. The annealed slab was rolled at 400°C by 15 passes to the final thickness of 1.1 mm with intermediate re-heating for 5–10 min after each rolling step for a stable rolling temperature of the slab. The rolled sheet was heat-treated at 350°C for 30 min to adjust the grain size to be appropriate for the CMWP fitting method with high grain statistics. Tensile samples with a gauge length of 18 mm and a thickness of 1.1 mm were machined along the sheet rolling direction (RD). *In situ* diffraction measurements were performed at the HEMS (High Energy Materials Science) beamline operated by the Helmholtz-Zentrum Hereon, P07B of PETRA III at the DESY (Deutsches Elektronen-Synchrotron) facility in Hamburg, Germany. A universal testing machine was installed at the beamline for tensile tests at room temperature with a initial strain rate of 1.0 × 10^−3^ s^−1^. The tensile sample was irradiated by hard X-rays with a beam size of 0.7 mm × 0.7 mm and an energy of 87.1 keV (wavelength = 0.142 Å). Debye–Scherrer diffraction rings were collected during the sample rotation of 105° in 3° steps using an area detector (PerkinElmer XRD 1621) with a fast read-out, at various strain values up to ɛ = 0.15. The measured data were processed about the transmutation of the diffraction pattern over selected omega and azimuth angles of the Deybe–Scherrer ring using the open source software *FiT2D* (Hammersley, 1998 ▸). Texture analysis was conducted with an orientation distribution function calculation from the measured pole figures using the open-source software toolbox *MTEX* (Bachmann *et al.*, 2010 ▸). For the X-ray line profile analysis, the diffraction patterns were evaluated by the CMWP fitting method. The diffraction patterns were integrated over a sample rotation of 24°, denoted as the omega angle, and an azimuth angle of 24° along the Debye–Scherrer ring at the section perpendicular to the loading direction (LD). The diffraction patterns were fitted by functions corrected with a background spline, an instrumental function obtained from an LaB_6_ standard sample, and a theoretical profile function (Ribárik *et al.*, 2001 ▸).

The microstructure characterization and determination of the global texture of the sheet were performed using optical microscopy and X-ray diffractometry on mechanically polished surfaces using an oxide polishing suspension. An electron backscatter diffraction (EBSD) measurement of the initial sample was carried out using a field emission gun scanning electron microscope (Zeiss, Ultra 55 installed with a Hikari detector, EDAX/TSL EBSD system), at an accelerating voltage of 15 kV and a working distance of 14 mm. The EBSD measurement area was 250 µm × 600 µm, measured in 0.4 µm steps, after standard sample preparation followed by electrolytic polishing at 16 V and −20°C for 60 s.

## 3. Results and discussion

**Figure 1:**
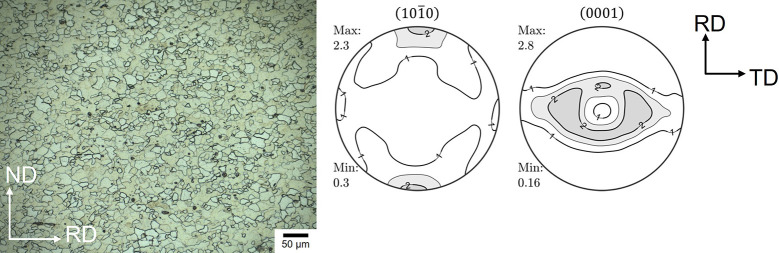
Optical micrograph and recalculated { 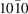 } and (0001) pole figures from laboratory X-ray diffraction of an annealed ZW10 sheet.

Fig. 1 ▸ shows the microstructure and texture of the rolled ZW10 sheet after recrystallization annealing at 350°C for 30 min. The sheet exhibits a relatively homogeneous microstructure with an average grain size of 11.4 µm, which provides sufficient grain statistics for the *in situ* diffraction study with a beam size of 0.7 mm × 0.7 mm at the synchrotron beamline. The annealed sheet has a weak texture with the basal poles tilted from the normal direction (ND) towards the transverse direction (TD) of the sheets with a relatively low basal pole intensity, *P*_max_ = 2.8 m.r.d (multiple of random distribution), compared with the conventional Mg alloys, *e.g.* AZ31 (Kaiser *et al.*, 2003 ▸).

**Figure 2:**
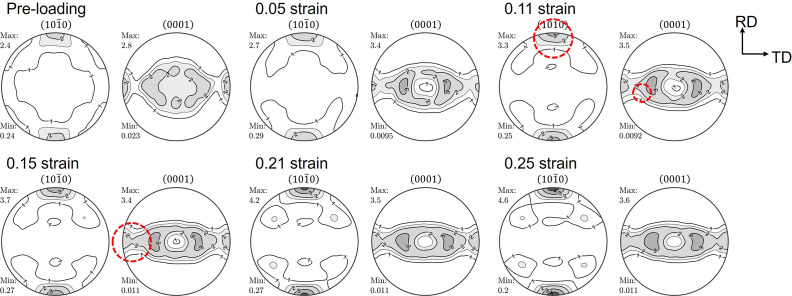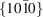 and (0001) pole figures measured at certain strains – ɛ = 0.05, 0.11, 0.15, 0.21 and 0.25 – by tensile loading along the RD of a ZW10 sheet. Levels: 1.0, 1.5, 2.0, 3.0, 4.0, 5.0 m.r.d. Red dashed circles: (0001) poles gradually broadened towards the TD. Blue arrow: symmetric position on the 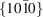 pole figure.

The texture developed during the tensile loading along the RD is shown in Fig. 2 ▸. With increasing strain, the ( 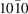 ) pole strengthens in the LD; concurrently, the (0001) poles gradually broaden towards the TD (perpendicular to the LD, red dashed circles in Fig. 2 ▸). The position of the maximum intensity of the (0001) poles is tilted away from the ND towards the TD, ±35° from the ND, and the six poles at the symmetric position on the { 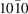 } pole figure are accordingly indicated (blue arrow in Fig. 2 ▸). In other words, the 〈 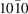 〉 fiber texture component along the LD developed during the tensile loading (Zhou *et al.*, 2020 ▸). A similar tendency of texture development during the tensile loading was reported for Mg alloys containing an RE element, *e.g.* Mg–Zn–Nd, having a weakened texture. During the tensile loading, such texture development is associated with the predominant activation of the prismatic 〈*a*〉 dislocation slip (Ha *et al.*, 2019 ▸, 2021 ▸).

**Figure 3:**
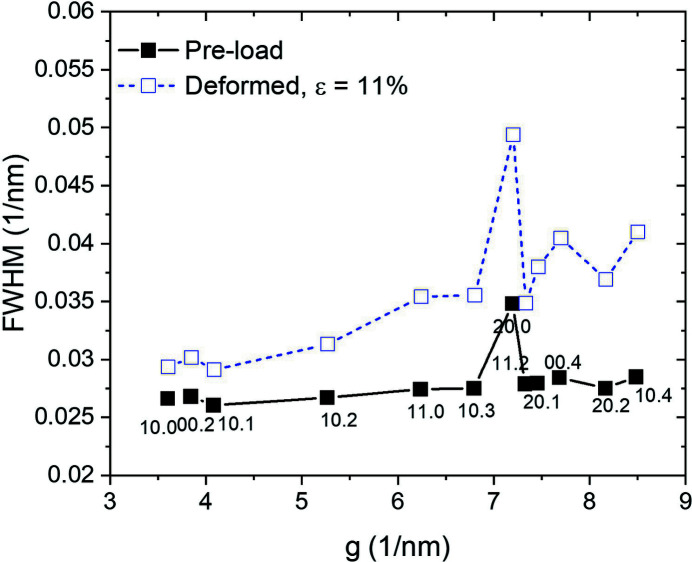
Williamson–Hall plot of the FWHM of diffraction peaks at ɛ = 0% (solid) and ɛ = 11% (open) of the ZW10 tensile sample (**g**: diffraction vector).

A WH (Williamson–Hall) plot allows identification of whether the deformation is mainly accommodated by dis­location slip or stacking faults such as twinning. In a WH plot, when the twinning activity is dominant during the deformation, the FWHM (full width at half-maximum) of the high-order diffraction peak shows a similar amount of broadening as that of its low-order peak, *e.g.* similar breadth of 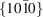 and 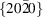 diffraction peaks. Contrarily, the FWHMs are gradually broadened according to the increase of the diffraction vector, **g**, when the dislocation slip plays the main role in the deformation modes. The WH plot of the sample examined in the present study shows a non-monotonic broadening of the FWHM for the deformed status with respect to the diffraction vector as shown in Fig. 3 ▸. This means that the strong strain anisotropy due to the dislocation slip causes broadening of the FWHM during tensile loading, which is important in the present study to check prior to conducting the whole diffraction pattern fitting using the CMWP method. Even if the twins play a role in the deformation of Mg alloys, the results of the WH plot indicate that the contribution of twinning is limited and mainly relevant during the early deformation stage. Therefore, the present study focuses on the evaluation of the dislocations activity, as the dominant deformation mode during the whole deformation, rather than twinning.

**Figure 4:**
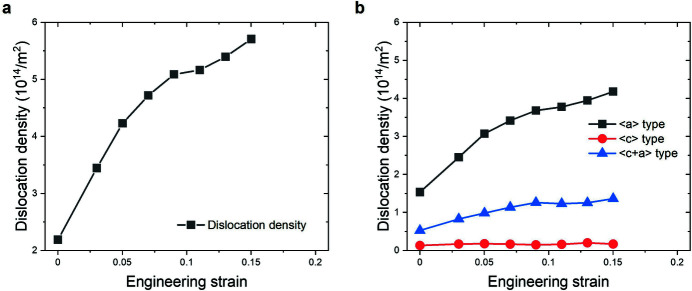
(*a*) Overall dislocation density and (*b*) dislocation densities with different Burgers vectors – 〈*a*〉, 〈*c*〉 and 〈*c*+*a*〉 – as a function of engineering strain obtained from the CMWP fitting method for a tensile deformed sample of ZW10.

**Figure 5:**
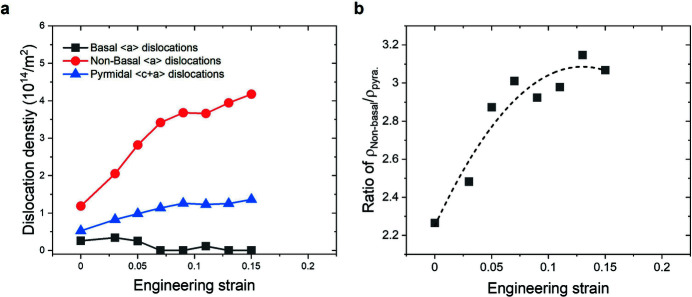
(*a*) Evolution of the dislocation densities of the basal, non-basal 〈*a*〉 and pyramidal 〈*c*+*a*〉 dislocations during tensile deformation of ZW10. (*b*) Ratio of non-basal 〈*a*〉 to pyramidal 〈*c*+*a*〉 dislocations.

The evolution of the overall dislocation density during the tensile deformation is shown in Fig. 4 ▸(*a*). It is apparent that the overall dislocation density increases with strain during the whole deformation, from approximately 2.2 × 10^14^ m^−2^ to 5.7 × 10^14^ m^−2^. This is in a good agreement with the anticipated finding of the WH plot (Fig. 3 ▸). The density ratio between the dislocations with different Burgers vectors – 〈*a*〉, 〈*c*〉, 〈*c*+*a*〉 types – is similar over the examined strain range. From the measured overall dislocation density, the 〈*a*〉 dislocations have a dominant fraction of about 70% and the 〈*c*+*a*〉 dislocations activity amounts to about 20%, while the 〈*c*〉 dislocations have the lowest fraction of less than 10%. The lowest activity of the 〈*c*〉 dislocations, with density less than 0.2 × 10^14^ m^−2^, agrees with previous studies (Yoo, 1981 ▸). Besides the overall dislocation density, an evaluation of the dislocations with different Burgers vectors possible in the hexagonal close-packed structure was conducted. A detailed explanation of this procedure can be found elsewhere (Máthis *et al.*, 2004 ▸; Dragomir & Ungár, 2002 ▸). It must be noted that it is difficult to distinguish the broadening effect of prismatic 〈*a*〉 from pyramidal 〈*a*〉 dislocations by X-ray line profile analysis (Ha *et al.*, 2021 ▸; Krajňák *et al.*, 2019 ▸), such that the sum of the densities is considered as the non-basal 〈*a*〉 dislocations in the present study. That is, the density evolutions of basal 〈*a*〉, non-basal 〈*a*〉 and pyramidal 〈*c*+*a*〉 dislocations with strain were evaluated [Fig. 5 ▸(*a*)]. The density of the basal 〈*a*〉 dis­locations remains at a low value of 0.5 × 10^14^ m^−2^, whereas the non-basal 〈*a*〉 and pyramidal 〈*c*+*a*〉 dislocations significantly increase with tensile deformation. The density of non-basal 〈*a*〉 dislocations reached the maximum value of about 4.2 × 10^14^ m^−2^, as the most activated dislocation, at ɛ = 0.15. It can be seen that the non-basal 〈*a*〉 dislocations contribute to the strain accommodation by having a ratio of more than two to three times that of the pyramidal 〈*c*+*a*〉 dislocations during the whole deformation [Fig. 5 ▸(*b*)].

**Figure 6:**
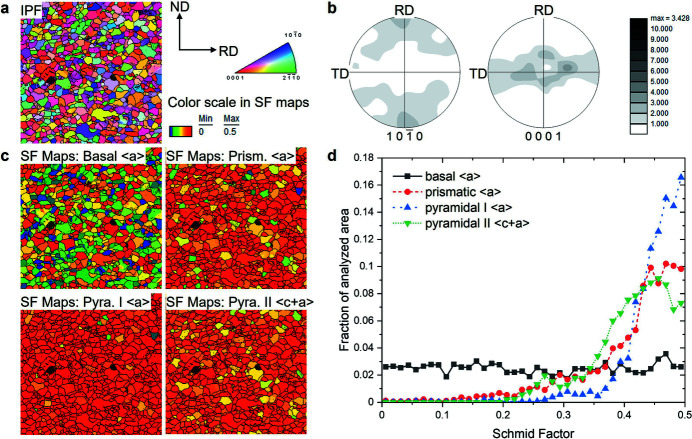
(*a*) EBSD orientation map, (*b*) 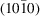 and (0001) pole figures, (*c*) color maps of Schmid factor (SF) analysis, and (*d*) SF fraction of basal, non-basal 〈*a*〉 and pyramidal 〈*c*+*a*〉 slip systems for the initial state of ZW10.

**Figure 7:**
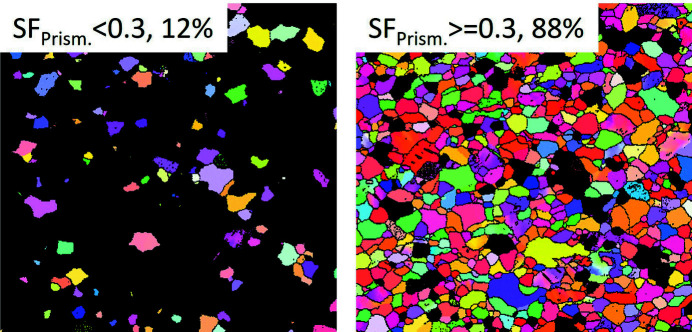
EBSD maps of SF values less than (left) and greater than (right) 0.3 for prismatic 〈*a*〉 slips.

EBSD inverse pole figure maps, recalculated pole figures and SF (Schmid factor) maps for the 〈*a*〉 and 〈*c*+*a*〉 dislocation slip systems of the annealed ZW10 sheet are presented in Fig. 6 ▸. The EBSD texture is similar to that measured using synchrotron radiation. In addition to the X-ray line profile analysis, the dislocations activities were complementarily examined by SF analysis of (0001)〈 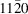 〉 basal 〈*a*〉, { 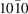 }〈 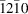 〉 prismatic 〈*a*〉, { 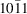 }〈 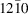 〉 pyramidal I 〈*a*〉 and { 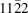 }〈 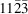 〉 pyramidal II 〈*c*+*a*〉 dislocation slip systems for tensile loading along the RD. It is clearly seen that the prismatic and pyramidal I 〈*a*〉 slip, as well as the pyramidal II 〈*c*+*a*〉 slip systems, have a large area with the SF in the range 0.4–0.5 [Fig. 6 ▸(*d*)]. Fig. 7 ▸ shows the grains with SF values of less than (left) and higher than (right) 0.3 for the prismatic 〈*a*〉 slip system. Most grains with an area fraction of 88% have favorable SF values for the non-basal slip systems, while the grains having a low SF value for the prismatic 〈*a*〉 slip system are favorable for the basal 〈*a*〉 slip.

**Figure 8:**
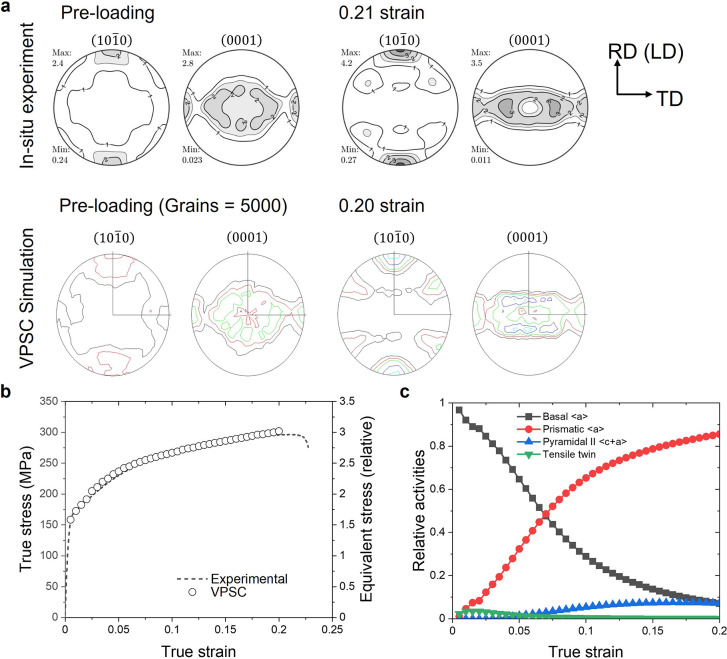
Comparison of experimental and simulated (*a*) textures at initial and 0.20 strains, (*b*) flow curves and (*c*) the simulated activities of the deformation modes during the tensile loading of a ZW10 sheet.

The reliability of the results obtained by the X-ray line profile analysis was also confirmed by the VPSC simulation. A detailed explanation of the VPSC model can be found elsewhere (Jain & Agnew, 2007 ▸; Tomé *et al.*, 1991 ▸). The parameters of the VPSC simulation, like the CRSS (critical resolved shear stress) values and the strain hardening of the individual slip based on Voce hardening (Tomé *et al.*, 1991 ▸; Bohlen *et al.*, 2007 ▸), were determined from fitting to the experimental tensile curve and texture evolution obtained from the *in situ* experiments. The texture measured at the initial state was reproduced using *MTEX* to an ensemble of 5000 grains (orientations) for the simulation. The parameters of the VPSC simulation that result in the best fit to the *in situ* experimental results are listed in Table 1 ▸. The deformation texture from the VPSC simulation agrees well with the *in situ* experimental results, *e.g.* (0001) poles broadened towards the TD and ( 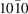 ) pole strengthened in the LD [Fig. 8 ▸(*a*)]. The activities of the deformation modes show a similar trend to those obtained from the CMWP analysis. The activity of the prismatic 〈*a*〉 dislocation slip, considered as non-basal 〈*a*〉 dislocations in the CMWP analysis, rapidly increases from the beginning of the deformation and remains as the main deformation mode, while the activity of the basal 〈*a*〉 slip system decreases with increasing strain. That is, the VPSC simulation results verify also that the deformation is mostly accommodated by the non-basal 〈*a*〉 dislocations [Fig. 8 ▸(*c*)]. The pyramidal II 〈*c*+*a*〉 dislocation slip shows a gradual increasing activity during the deformation. It is also in good agreement with results of the CMWP evaluation. It is known that the CRSS value of the basal 〈*a*〉 slip system is considerably lower than that of non-basal slip systems in Mg alloys. Jain & Agnew (2007 ▸) reported that the ratio between the CRSS values for the slip systems in conventional Mg alloy, AZ31, is τ_basal_:τ_prism_:τ_pyra_ = 1:3.2:5. The CRSS ratio for the ZW10 is to be amended to τ_0(basal)_:τ_0(prism)_:τ_0(pyra)_ = 1:2.3:2.8 in the present study. Note that the τ_0_ and τ_1_ values used in the VPSC simulation, in Table 1 ▸, represent the relative CRSS values of the corresponding deformation mode. These results can be interpreted as the increase in the CRSS value of the basal 〈*a*〉 dislocation slip, or the decrease in the CRSS values of the non-basal dislocations slip in the ZW10 alloy.

The altered CRSS on the slip systems is related to the addition of alloying elements – Zn and Y in this study. It is well documented that the addition of RE elements decreases the CRSS ratio of non-basal 〈*a*〉 to basal 〈*a*〉 slip systems (Bohlen *et al.*, 2007 ▸), and this tendency is promoted by the simultaneous addition of Zn (Basu & Al-Samman, 2014 ▸; Ha *et al.*, 2021 ▸). It is actually expected that the basal 〈*a*〉 slip system shows a high activity due to a lower CRSS value in comparison with other slip systems, especially at the beginning of plastic deformation. However, the CMWP results do not show a high activity of basal 〈*a*〉 dislocation slip systems [see, for example, Fig. 5 ▸(*a*)]. A slight increase in basal 〈*a*〉 dislocation slip appears at the beginning of the plastic deformation, but it is not so noticeable, while the activities of non-basal 〈*a*〉 dislocations slip significantly increase. The higher activities of the non-basal slip systems even at the beginning of the plastic deformation can be understood to be as a result of the higher SF of the non-basal 〈*a*〉 dislocations slip [Fig. 6 ▸(*d*)]. Moreover, the advantage in CRSS and SF of non-basal dislocations of the ZW10 sheet assures high activities even at high strain. The texture evolution of the *in situ* experiment shows that the (0001) poles gradually broadened towards the TD with increasing strain (Fig. 2 ▸). Although the developed textures slightly differ between the VPSC simulation and *in situ* experiment, their trends of texture evolutions are similar. This is related to the higher activation of the non-basal dislocations slip, not the basal 〈*a*〉 dislocation slip, which was verified by the VPSC simulation. In summary, X-ray line profile analysis using the CMWP fitting method can reveal reasonable results on the change in dislocation density and their activities, which are currently widely analyzed by electron microscopy observation or computer simulation.

## 4. Conclusion

The evolution of dislocation densities with different Burgers vectors was studied using the CMWP evaluation of the *in situ* diffraction during tensile loading of an Mg–Zn–Y (ZW10) alloy sheet, and the results regarding active deformation modes were further analyzed in comparison with EBSD measurements and VPSC simulation.

WH plots showing the broadening of the FWHM with strain indicate that the dislocation slip plays an important role in deformation accommodation of the ZW10. The overall dislocation densities increase with increasing deformation, from approximately 2.2 × 10^14^ m^−2^ to 5.7 × 10^14^ m^−2^.

The density of basal 〈*a*〉 dislocations remains at a low value of 0.5 × 10^14^ m^−2^, whereas the non-basal dislocations significantly increase with tensile deformation, especially non-basal 〈*a*〉 dislocations. The texture developments corresponding to the ( 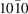 ) pole strengthening along the LD and the (0001) poles broadening towards the TD can be understood as a result of the higher activities of non-basal 〈*a*〉 dislocations.

EBSD analysis shows that a large area fraction of the annealed sheet has an SF value within the range 0.4–0.5 for the prismatic and pyramidal I 〈*a*〉 slips, as well as pyramidal II 〈*c*+*a*〉 slip systems. Moreover, the VPSC simulation also shows that the deformation is mostly accommodated by the dominant non-basal 〈*a*〉 slip systems and pyramidal II 〈*c*+*a*〉 slip system. These results indicate that the CMWP analysis is considerably reliable for evaluating the deformation modes and their behavior in Mg alloy sheets.

## 5. Data availability

The raw/processed data required to reproduce these findings cannot be shared at this time as the data also form part of an ongoing study.

## Figures and Tables

**Table 1:** Voce hardening parameters of the individual deformation mechanisms fit to the behavior of the examined sheets, ZW10 (τ_0_ and τ_1_ represent the relative CRSS values of the corresponding deformation mode)

Deformation mode	τ_0_	τ_1_	θ_0_	θ_1_
Basal 〈*a*〉	0.7	0.3	100	2.0
Prismatic 〈*a*〉	1.6	0.4	30	0.5
Pyramidal 〈*c*+*a*〉	2.0	1.0	600	0.8
Tension twin	1.2	0	0	0
